# Microscopic detection and genetic characterization of schistosome eggs within cervicovaginal lavage sediments from cases of female genital schistosomiasis

**DOI:** 10.1017/S0031182025100656

**Published:** 2025-12

**Authors:** J. Russell Stothard, Bright Mainga, Dingase Kumwenda, Alice Chisale, Tereza Nchembe, Christine Rice, Lilly Atkins, Guillery Deles, Sam Jones, Lucas J. Cunningham, Peter Makaula, Sekeleghe A. Kayuni, Janelisa Musaya

**Affiliations:** 1 Department of Tropical Disease Biology, Liverpool School of Tropical Medicine, Liverpool, UK; 2 Neglected Tropical Diseases Group, Malawi Liverpool Wellcome Programme, Kamuzu University of Health Sciences, Queen Elizabeth Central Hospital Campus, Blantyre, Malawi; 3 Laboratory Department, Mangochi District Hospital, Mangochi, Malawi; 4 Obstetrics and Gynaecology Department, Queen Elizabeth Central Hospital, Blantyre, Malawi; 5 Pathology Department, School of Medicine and Oral Health, Mahatma Gandhi Campus, Blantyre, Malawi

**Keywords:** colposcopy, Malawi, menstrual hygiene management, *Schistosoma haematobium*, *Schistosoma mattheei*, urogenital schistosomiasis

## Abstract

Control of female genital schistosomiasis (FGS) has gained significant international attention, driven in part, as a newly appreciated underlying aetiological risk factor for HIV, HPV and cervical dysplasia. Whilst diagnosis and clinical staging of FGS typically relies upon colposcopy, alternative methods of incrimination have grown, particularly upon application of PCR diagnostic assays that detect schistosome DNA within tissue biopsy, genital (self-)swab and/or cervicovaginal lavage (CVL). With regard to the latter, we present novel evidence that microscopy alone of CVL sediments can be sufficient to incriminate FGS and CVL sediment provides an original source of (viable) schistosome eggs and miracidia for later genetic analysis. Upon a pilot examination of 55 adult women from Malawi with previously proven urogenital schistosomiasis by egg-patent urine microscopy, 25.5% (95% CI = 14.7–39.0) were found to have schistosome eggs within CVL, with one woman having more than 50 eggs observed. After praziquantel treatments and upon re-examination one year later, the prevalence of egg-patent CVLs reduced to 14.5% (95% CI = 6.5–26.7) although the same woman again presented with more than 50 observable eggs. Molecular DNA analysis by real-time PCR of extracted DNA from CVL sediments and CVL hatched miracidia (and eggs) revealed the dominance of *Schistosoma haematobium* within the samples, noting a fifth with *Schistosoma mattheei* co-infections and the singular presence of a putative *S. haematobium* × *mattheei* hybrid miracidium. Viable schistosome eggs shed from cervicovaginal surfaces likely represent a minor environmental transmission route, thus promoting secure menstrual hygiene management is needed.

## Graphical abstract

**Figure:**
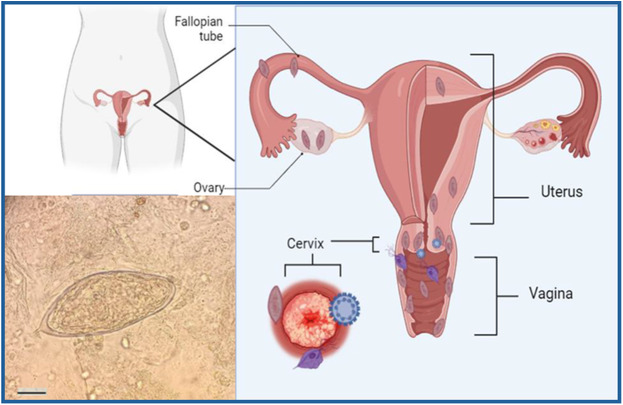

## Introduction

Parasitological diagnosis of human schistosomiasis by light microscopy remains a foundational tool for individual detection and community monitoring of urogenital and intestinal schistosomiasis (Buonfrate et al., 2025). With regard to urogenital schistosomiasis, collection of schistosome eggs within mid-day urine, by centrifugation or by syringe filtration methods for later viewing and enumeration under the compound microscope, is routinely used in primary health care, disease mapping initiatives and anthelminthic drug efficacy spot-checks (WHO, 2013, 2022). Female genital schistosomiasis (FGS) is a gender-specific manifestation of urogenital schistosomiasis, strongly linked to several stigmatizing and detrimental effects on a woman’s sexual and reproductive health; it is typically caused by chronic infection with *Schistosoma haematobium* (Lamberti et al., 2024a), though other species of schistosome (Christinet et al., 2016), and latterly those schistosomes of hybrid origin(s) (Kayuni et al., 2024) have been incriminated.

Currently across Africa, several tens of millions of women have (undiagnosed) FGS (Bustinduy et al., 2022; Umbelino-Walker et al., 2023), alongside many travel-related cases reported across the world (Helling-Giese et al., 2024; Marchese et al., 2025), which have raised international public health attention on its prevention and control (Lamberti et al., 2024a). Despite its first report over a century ago (Madden, 1899), FGS in sub-Saharan Africa is a newly appreciated underlying aetiological risk factor for HIV, HPV and cervical dysplasia (Engels et al., 2020; Umbelino-Walker et al., 2023; Sturt et al., 2025). Madden’s seminal 1899 observation in Cairo is still relevant today for he described a singular case of vaginal and not urinary schistosomiasis within a young Egyptian woman; numerous schistosome eggs were demonstrated within a papillomatous vaginal mass surgically removed yet no schistosome eggs were detected in her urine despite repeat sampling (Madden, 1899). From best epidemiological evidence today, the presence of schistosome eggs in urine is not a strong predictor of clinical FGS nor its level of severity, particularly in the upper genital tract where such internal organs remain secluded and largely overlooked without recourse to abdominal surgery or post-mortem (Shennan and Gelfand, 1971; Wright et al., 1982; Bustinduy et al., 2022; Umbelino-Walker et al., 2023; Lamberti et al., 2024a).

Contemporary methods to diagnose and clinically stage FGS have benefited from the use of point-of-care colposcopy, where lower genital tract damage, *viz*. cervicovaginal lesions, are viewed directly then compared against those within the WHO pocket atlas for health care professionals (WHO, 2015; Martinez et al., 2024). In Malawi, for example, colposcopy was first used during the mid-1990s to better appreciate the burden of FGS in women (Kjetland et al., 1996) which had been noted previously upon histopathology (Wright et al., 1982). Gynaecological inspections with colposcopy were augmented with parasitological examination by urine microscopy, as well as inspection for schistosome eggs within cervicovaginal biopsies by simple crushing of excised tissues between two glass slides (Kjetland et al., 1996). More recently, the MORBID study undertaken in Malawi has drawn new focus and public health concern on an extensive but underreported widespread burden of FGS (Lamberti et al., 2024b).

While such tissue biopsy methods can provide a definitive parasitological diagnosis, they are invasive, uncomfortable and until the biopsy wound site has healed, carry a later increased risk of infection, particularly HIV, for the patient (Kjetland et al., 2012; Bustinduy et al., 2022). Less invasive sampling methods such as retrieval and inspection of cervicovaginal lavage (CVL) offer an alternative (Kjetland et al., 2009) but to the best of our knowledge, CVL sediments have not yet been formally inspected by microscopy for schistosome eggs directly. Nevertheless, analysis of CVL sediments has been a convenient, routine specimen for molecular diagnostic assays for several parasites and pathogens of the genital tract (Delany et al., 2008), inclusive of schistosomes (Kjetland et al., 2012; Patwary et al., 2021). For example, Kjetland et al. (2009) first used real-time PCR with a schistosome-specific probe on analysis of CVL sediments collected during outpatient clinic sampling in Zimbabwe. More recently, to expand community-based surveillance of FGS in Zambia, a similar molecular diagnostic assay has been applied on self-sampled genital swabs (Sturt et al., 2020), alongside further use of CVL sediments with recombinase polymerase amplification-based assays (Archer et al., 2022).

In this report, we take advantage of the FGS sub-study within the larger HUGS-*Hybridisation in UroGenital Schistosomiasis* investigation to pilot assess the potential use of CVL sediments to augment parasitological and clinical gynaecological examinations. Specifically, could schistosome eggs be directly detected by light microscopy within CVL sediments and could viable eggs be hatched to harvest individual miracidia for later genotyping of schistosome species- and/or hybrid-specific diversity?

## Materials and methods

### Study site locations and participant recruitment

**Figure 1.**
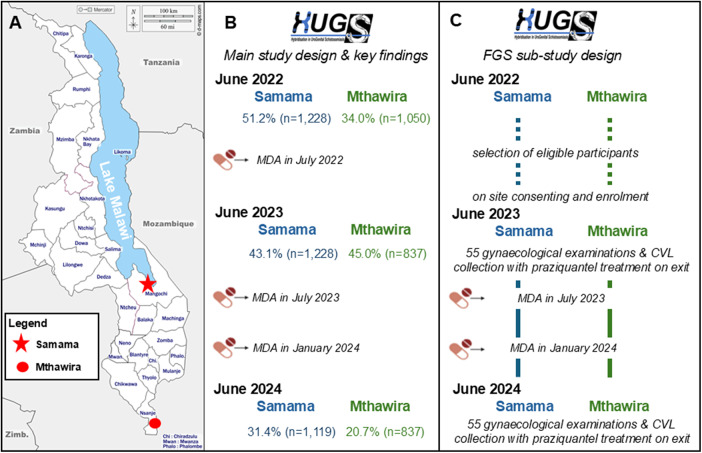
Schematic map of the study sites with outline activities of the main HUGS investigation and FGS sub-study. (A) District map of Malawi denoting the two study villages, Samama in Mangochi District and Mthawira in Nsanje District. (B) Outline of activities of the main HUGS investigation commencing with baseline survey in June 2022. The prevalence of schistosome egg-patent urines is shown at each inspection time point. Both communities received mass drug administration praziquantel in line with national control programme governmental procedures. (C) Outline of activities of the HUGS FGS sub-study which started recruitment in late May 2023 with two gynaecological inspections in June 2023 and June 2024. Of note, all FGS participants were offered praziquantel upon exit of the gynaecological examination and would have also received biannual mass drug administration of praziquantel before the June 2024 follow-up. A final clinical review with dissemination of findings for the FGS sub-study cohort took place in January 2025. Note MDA is ‘mass drug administration’ with praziquantel using local community drug distributors within each village.

This FGS sub-study was conducted in June 2023 and in June 2024. This sub-study was formally set within the larger HUGS investigation to monitor the molecular epidemiology of hybrid schistosomes, using urine sampling and examination, in two sentinel study communities in Mangochi District at Samama (S 14.418767^0^, E 35.220985^0^) and in Nsanje District at Mthawira (S 16.849802^0^, E 35.290041^0^), Figure 1A. Key demographical and summary parasitological data are reported by Makaula et al. (2025), consisting of a closed cohort study of 2,500 participants recruited at baseline, initially aged from 2 to 65 years old, in June 2022 who were re-examined upon two later annual follow-ups, when the FGS sub-study took place. The larger study completed in January 2025, Figure 1B. These two sites were chosen as locations were schistosome hybrid infections were originally found in the urine of several primary school children (Webster et al., 2019). Samama is located on the near shoreline of Lake Malawi bordering the western side of the mouth of the Upper Shire River while Mthawira is located some 275 km southwards adjacent to the western banks of the Lower Shire River, Figure 1A.

Upon the wider HUGS baseline study undertaken in June 2022, the overall egg-patent prevalence of urogenital schistosomiasis in each community was 51.2% (Samama, *n* = 1,228) and 34.0% (Mthawira, *n* = 1,050), after which each community received mass drug administration with praziquantel, Figure 1B. Upon annual community cohort follow-up in June 2023, the egg-patent prevalence was 43.1% (Samama, *n* = 1,228) and 45.0% (Mthawira, *n* = 837). In light of these high levels, despite annual treatment, biannual mass drug administration took place in July 2023 and then in January 2024, with a further final follow-up in June 2024, when the prevalence had reduced to 31.4% (Samama, *n* = 1,119) and 20.7% (Mthawira, *n* = 837), Figure 1B, (Makaula et al., 2025).

Using parasitological information from the June 2022 community baseline survey, all adult women aged between 18 and 55 years old, who had egg-patent schistosomiasis in urine (and several with non-*S. haematobium* schistosome DNA signatures within extracted DNA from their urine filter), were identified for potential inclusion into the FGS sub-study, Figure 1C. The main rationale of the FGS sub-study was to incriminate zoonotic (*S. mattheei*) or hybrid (*S. haematobium* × *mattheei*) schistosome species in genital tract disease of women (Stothard et al., 2025).

In June 2023 and June 2024, a combined total of 78 adult women agreed to undergo gynaecological examination, of which a complete data set from 55 individuals was obtained. This was following on from further community sensitization(s) with stringent local procedures recruiting into the FGS sub-study. Women were provided with FGS-specific patient information sheets and consent forms in local languages of Chichewa, Chiyao and Chisena, alongside one-to-one dialogue with the gynaecological team (D.K., A.C. & T.N.), if eligible. Gynaecological examinations with colposcopy, used a MobileODT EVA COLPO (FemTech, Tel Aviv, Israel) as overseen by two trained midwives (A.C. and T.N.) with supervision from the consultant gynaecologist (D.K.), Figure 1C. During gynaecological examination, CVL samples were first obtained then colposcopy was undertaken. If deemed necessary, genital tissue biopsies were performed after acetic acid staining of cervicovaginal surfaces. Detailed gynaecological findings, alongside patient responses to a signs and symptoms clinical questionnaire, exploring general sexual and reproductive health, will be presented elsewhere (Kumwenda et al., In review). Here, only essential information from colposcopy directly related to CVL sediment comparisons is reported.

All participating women provided a mid-morning urine sample which was examined the same day for schistosome eggs upon standard syringe filtration of a 10 ml sub-sample, following WHO guidelines (WHO, 2022). At the end of the gynaecological examination with an exit consultation, each patient was provided with an appropriate dose of praziquantel (40 mg/kg) in 600 mg (half-) tablets of Cesol® (Merck-KGa, Germany) to later take at home with food, Figure 1C, as recommended by the Malawi national control programme and by WHO (WHO, 2022).

### Collection of cervicovaginal lavage and processing for microscopy

Collection of CVL took place with the patient lying supine on a standard gynaecological bed, with appropriate leg support. With metal speculum placed *in situ* and using a plastic syringe, 10 ml of buffered normal saline (pH 7.4) was flushed against the vaginal walls, fornixes and cervix. Using a 2 ml disposable pastette, this solution was harvested into a 15 ml Falcon tube for processing later the same day within the field laboratory.

Each CVL lavage then underwent centrifugation at 6,000 rpm for 5 min within a benchtop centrifuge. The supernatant was decanted off, visually inspected for blood against a white card background, then tested for cryptic blood using Multistix 10SG reagent strips (Siemens Healthcare Limited, Camberley, UK). The remaining pellet of CVL sediment was resuspended into 0.5 ml of normal buffered saline. Approximately half of this resuspension was applied onto a microscope glass slide, with a double glass cover slip overlaid, before viewing under the compound microscope with enumeration of any schistosome eggs present, as classified into three intensity of infection categories: 1–9, 10–49 and ≥ 50 eggs. The remaining resuspension was transferred into a 2 ml screw-top tube, containing 1 ml of absolute ethanol, for subsequent DNA analysis.

When a schistosome egg was seen by microscopy, attempts were made to hatch the egg to release the miracidium on the same day. To do so, the glass slide was placed in a standard plastic Petri dish containing 20 ml of mineral water. Using fine forceps, the glass cover slip was removed and rinsed in the mineral water then discarded. The Petri dish with the glass slide therein was then placed under a dissecting microscope with a black background directly underneath, then illuminated from the side with a strong LED headtorch. A dark plastic disk was also placed on top of the Petri dish to help concentrate the illumination of the side light to encourage swimming miracidia to assemble towards the front edge of the Petri dish. After 15–30 min incubation at room temperature, the Petri dish was inspected at 20× magnification to view miracidia. If present, these were harvested in 2.5 µl and then spotted by pipette onto Whatman Flinders Technology Associates (FTA) indicating cards (GE Healthcare Life Sciences, https://www.gehealthcare.com/products/life-sciences). Should no miracidium be seen, the Petri dish was further inspected for schistosome eggs and if seen were harvested in 2.5 µl and then spotted by pipette onto Whatman FTA indicating cards.

### Genetic analysis of cervicovaginal lavage sediments

All CVL sediments in ethanol were transferred to the LSTM for genomic DNA extraction and application of real-time PCR with species- and hybrid-specific typing assays (Cunningham et al., 2024; Kayuni et al., 2024). Total DNA from CVL sediments was extracted using Qiagen DNeasy Blood and Tissue Kit (Citylabs 2.0, Hathersage Road, Manchester, UK). Genomic DNA of miracidia (and eggs) was extracted by alkaline elution from circular 2 mm diameter punches of individual spots on the Whatman FTA card; briefly, punches were incubated at room temperature for 5 min in 0.1 M NaOH, 0.3 mM EDTA (pH 13.0) then pH neutralized with equal volume of 0.1 M Tris-HCL (pH 7.0), pulse vortexed, with further incubation at room temperature for 10 min before a 3 µl aliquot of the solution was transferred for immediate use in real-time PCR or the remainder placed in short-term storage at 4–8^o^C (Cunningham et al., 2024). All CVL sediments and miracidia (and eggs) underwent a preliminary real-time PCR inspection with a schistosome genus-specific Taqman® assay, considering Ct values of 35 cycles or less as positive, then if found considered positive were re-screened with a novel species-specific high-resolution melt (HRM) profiling assay inspecting both nuclear and mitochondrial target loci, as described by Cunningham et al. (2024).

### Analysis of urine, cervicovaginal lavage and molecular information

After patient record checking and data verification against consent forms across 2023 and 2024 FGS surveys, information from a total of 55 participants was available for analysis and cross-tabulations for urine, CVL, molecular assays and colposcopy parameters.

### Research ethical review

Written informed consent to participate in the FGS sub-study was obtained from all of the eligible participants, either as signature or as thumbprint, as well as verbal assent immediately prior to the patient’s gynaecological examination. Both individual privacy and patient confidentiality were maintained throughout, inspections were undertaken within a designated field clinic with specific examination room where only female clinical staff were present.

Ethical approval for the HUGS study was granted by the College of Medicine Research Ethics Committee (COMREC), Kamuzu University of Health Sciences (KUHeS), Malawi, (Approval number: P.08/21/3381) and the LSTM Research Ethics Committee (LSTM REC) in the United Kingdom (registration number: 22-028), with material transfer agreements for international shipment of CVL sediments and FTA samples to the LSTM laboratories.

## Results

### Egg-patent urogenital schistosomiasis and analysis of cervicovaginal lavage

The June 2022 HUGS community baseline study detected urine-egg patent prevalence in adults of 16.8%; only women with urine egg-patent infections were eligible to be enrolled into this FGS study, therefore the prevalence of egg-patent urine was 100.0% but with associated infection intensities of: 1–9 (57.8%), 10–49 (34.4%) and ≥ 50 (7.8%), eggs per 10 ml. After annual mass drug administration and upon reinspection in June 2023, the overall prevalence of urine egg-patent infections within these women was now 34.4%, with the prevalence of infection intensities of: 1–9 (54.6%), 10–49 (31.8%) and ≥ 50 (13.6%). After biannual mass drug administration, the overall prevalence of urine egg-patency in the June 2024 reinspection was 12.9%, with infection intensity prevalences of: 1–9 (55.6%), 10–49 (33.3%) and ≥ 50 (11.1%), Table 1.

**Table 1.** The prevalence of urogenital schistosomiasis and female genital schistosomiasis at each survey for the 55 participants, where all data were available, as measured by microscopy for eggs, molecular DNA and visual colposcopy.

	Observed prevalence during FGS survey	
Diagnostic assessment	June 2023 (95% CI)	June 2024 (95% CI)
Egg-patent urine	34.4% (22.2−48.6)	12.9% (5.3−24.5)
Egg-patent CVL sediment	25.5%^y^ (14.7−39.0)	14.5%^y^ (6.5−26.7)
DNA patent CVL sediment	50.9% (37.1−64.7)	32.7% (20.7−46.1)
Any visual FGS-related lesion^*^	52.7% (38.8−66.4)	52.7% (38.8−66.4)

* The proportions of homogenous yellow sandy patches, grainy sandy patches, abnormal blood vessels, rubbery papules and ‘other’ slightly differed between surveys.

y Annual reduction in egg-patent CVL prevalence of 57.1% between June 2023 and June 2024 was non-significant (Chi-square = 1.37, *P* > 0.10).

**Figure 2.**
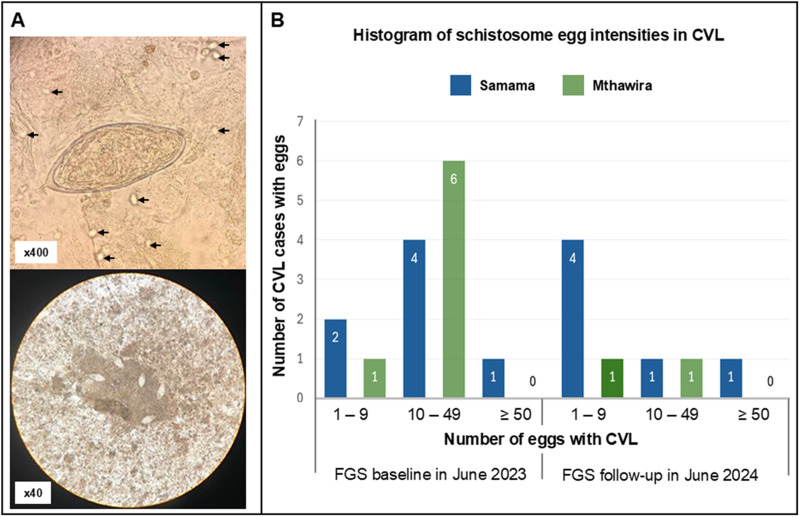
Illustrative images of schistosome eggs seen by microscopy and the occurrence of infected cases at FGS baseline (June 2023) and annual follow-up (June 2024). (A) *Top* – a viable egg of *S. haematobium*, with active flame cells, as seen at 400× magnification within the CVL. Motile trophozoites of *Trichomonas vaginalis* are also seen within this field of view, denoted by arrows; *bottom* – several eggs of *S. haematobium* seen within a single field of view at 40× magnification lodged within a sloughed-off raft of mucosal cellS. (B) Histogram column chart of the of the number of patients with schistosome eggs within their CVL, in categories of increasing infection intensity of: 1–9, 10–49 and ≥ 50 eggs within CVL sediment, at FGS baseline and annual follow-up.

Across the two surveys in June 2023 and June 2024, the age categories of this sub-study group of women were as follows: 18–25 yo (50.0%), 26–32 yo (21.2%), 33–39 yo (21.2%) and 40 + yo (7.6%). A total of 110 CVL matched sediments where available for analysis in which schistosome eggs were seen in 22 of these. All observed eggs were of typical size and shape for *S. haematobium*, many of these at 400× magnification were seen to be viable with flame cells active or with the miracidium twitching or moving within its egg-shell, Figure 2A.

The intensity of shed eggs within CVL exceeded a count of ≥ 50 on two occasions, though from the same individual across the surveys, where on first view a raft of schistosome eggs was easily seen at 40× magnification as embedded in sloughed-off mucosal tissue, Figure 2A. The egg-patent intensity category and village origin of positive CVL sediments are shown in Figure 2B. In June 2023, overall egg-patent prevalence of schistosomiasis in CVL sediment was 25.5%, then in June 2024 reduced to 14.5%, a percentage reduction of 57.1% which was non-significant (Chi-square = 1.37, *P* > 0.10), Table 1. In June 2023, frank or visible blood was seen in 16.4% CVL lavage with analysis by reagent strips revealing 57.4% with non-visible or cryptic blood. In June 2024, frank or visible blood was seen in 12.9% CVL lavage with analysis by reagent strips revealing 49.0% with non-visible or cryptic blood. At magnifications of either at 100× or 400×, motile trophozoites of *Trichomonas vaginalis* could be seen, Figure 2A.

### Analysis of cervicovaginal sediments, miracidia and colposcopy

**Figure 3.**
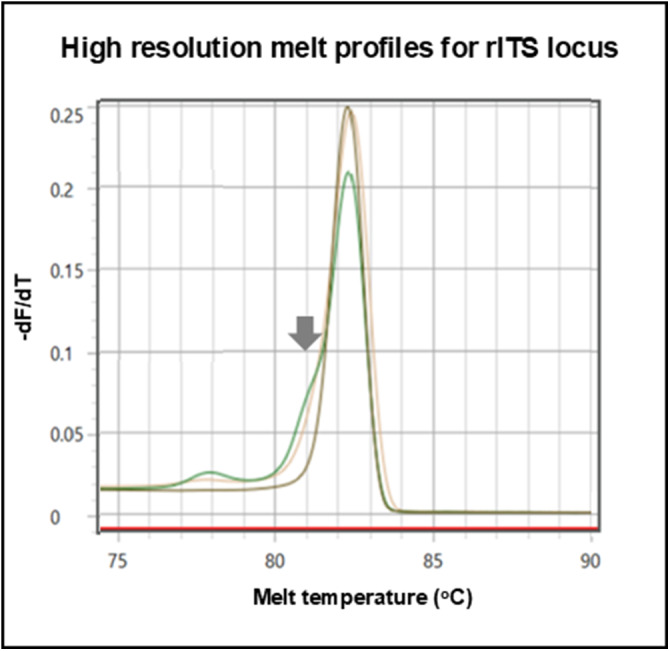
Application of real-time PCR high resolution melt assay detects species-specific variation within the nuclear ribosomal internal transcribed spacer (rITS) locus. The grey arrow depicts a melting curve ‘shoulder’ of in the green (CVL sediment) and yellow (miracidium) chromatograms indicative of mixed *S. haematobium* and *S. mattheei* DNA signatures, whereas the grey chromatogram is reference genomic DNA from a *S. haematobium* control sample.

Using a genus-specific real-time PCR assay, noting that Ct values of more than 35 cycles were considered negative, the prevalence of schistosome DNA within the CVL sediment in June 2023 and June 2024 was 50.9% and 32.7%, respectively. The lowest Ct value observed was at 18 cycles, corresponding to a sample where ≥ 50 eggs were seen. Using a species-specific HRM assay genetic diversity was detected with the nuclear locus assay, across the surveys, from 11 CVL sediments, upon having evidence of a mixed melt peak with characteristic minor ‘shoulder’, Figure 3, which most likely represented a mixed species genital infection with dominant *S. haematobium* and *S. mattheei*, though the presence of a hybrid *S. haematobium* × *mattheei* is also plausible. Upon HRM analysis with mitochondrial loci assays, only *S. haematobium* was judged present.

Across the two surveys, it was possible to hatch miracidia from CVL sediments, harvesting 163 miracidia onto FTA cards from a total of 9 women. Using real-time PCR with HRM assays for both mitochondrial and nuclear loci, 95 miracidia yielded genotyping data, an amplification success rate of 58.2%. Using a species-specific HRM assay, no variation was again observed with the mitochondrial loci, all being *S. haematobium*, but using the nuclear assay, a mixed chromatogram peak with characteristic ‘shoulder’ was detected for one miracidium in the June 2024 survey, Figure 3. The latter is indicative of a putative *S. haematobium* × *mattheei* hybrid.

The prevalence of any FGS lesion noted by colposcopy in June 2023 was 52.7% with the following visualized cervicovaginal surface sequelae classified as: homogenous yellow sandy patches (50%), grainy sandy patches (17%), abnormal blood vessels (14%), rubbery papules (5%) and ‘other’ (14%), as graded against the WHO pocket atlas imagery. Upon reinspection in June 2024 the prevalence of any FGS lesion remained unchanged but the percentage of cervicovaginal lesions by classification slightly differed: homogenous yellow sandy patches (50%), grainy sandy patches (19%), abnormal blood vessels (8%), rubbery papules (7%) and ‘other’ (16%), Table 1.

## Discussion

Our novel examination of CVL sediments by light microscopy, within the HUGS FGS sub-study, has firmly revealed that viable schistosome eggs and miracidia can be detected, in various levels of infection intensities, during both examinations separated by a calendar year, Figure 2. In June 2023, about a quarter of study participants were shown to have a variety of infection intensities in CVL sediments then, in June 2024, the overall prevalence reduced roughly in half, falling to 14.5%, Table 1. Though this is a welcome reduction through time, despite having access to two to three praziquantel treatment(s), some women continued to shed viable eggs in CVL, with a singular woman having an infection intensity of over 50 eggs, a noteworthy concern.

The prevalence of such a shed egg intensity, in a context of elimination of schistosomiasis as a public health problem, and more often arising from urine analysis discussions, is prominent since such infections should not exceed 1% within a sampled population (WHO, 2022). Here, even within a single CVL sediment, it clearly has on two occasions although the samples were from the same person separated by a calendar year. Across the study population in June 2024, approximately a seventh of women surveyed remained with egg-patent urine infections with about a tenth of these having heavy infection intensities, Table 1. In light of CVL findings, it is not well-known how successful such urine and non-urine shed eggs, i.e. from the woman’s cervicovaginal surfaces, would be in reaching the environment to infect freshwater snails, as once speculated by Shennan and Gelfand (1971) over 50 years ago. This suggestion, bolstered with the evidence presented here, helps to solidify better public health connections between promotion of environmentally secure menstrual hygiene management and those with FGS (Stothard et al., 2020b). Today, water, sanitation and hygiene and behavioural change interventions in sub-Saharan Africa (Torres-Vitolas et al., 2023) need to better address this special contaminating role, even if considered a ‘minor’ environmental transmission route (Shennan and Gelfand, 1971). The latter likely arises from women’s closer water contact behaviours from increased hygienic cleaning needs, regular washing of soiled underwear/clothes and inappropriate discard or cleaning of current menstrual hygiene products. Taken as a whole, each has the potential to release viable schistosome eggs, and hence miracidia into freshwater transmission, using non-urine/faecal dissemination routes.

The observation of CVL egg-patent prevalence of 14.5% in June 2024 is very solid evidence that even if praziquantel treatments were initially effective, these were beneficial only within a short-term perspective, like in other known urogenital transmission ‘hotspots’ (Sang et al., 2014; Privat et al., 2023). In our village setting of southern Malawi, from general community cohort analysis of urine egg-patent infections, it is clear that local reinfection risk is substantial across all diagnostic measures even after biannual mass drug administration, Table 1. For example, at Samama the reduction in general community egg-patent urine prevalence between 2023 and 2024 was 43.1% to 31.4%, while at Mthawira was 45.0% to 20.7% (Makaula et al., 2025). Worthy of mention here is that the HUGS investigation has been quarterly monitoring and repeat sampling freshwater snails locally and during this period has often found populations of *Bulinus* shedding schistosomes in Mangochi District and, to a lesser extent, in Nsanje District.

Mindful of the well-known difficulties in FGS diagnosis and clinical staging in individuals when no single test or method can adequately fulfil all diagnostic needs (Lamberti et al., 2024a), we surmise that microscopic analysis of CVL sediment, as a standalone approach, will never incriminate all FGS cases sufficiently. This was recently evidenced and elaborated upon by Kayuni et al. (2024) on a problematic case of FGS in Nsanje District. It is therefore unsurprising to see gross differences, from diagnostic discordance, in estimated prevalence of FGS from CVL microscopy, molecular CVL analysis and clinical colposcopy, Table 1. Indeed, any parasitological method could never be a precise diagnostic proxy of the actual clinical morbidity from FGS where cervicovaginal vascular remodelling, friable mucosa, local inflammation and granulomatous fibrosis occur, all of which are largely mediated by host-specific immunopathological dynamics, notwithstanding an individual’s access to praziquantel treatment(s) through time (Kjetland et al., 2012; Kayuni et al., 2024). Nonetheless, although clearly diagnostically insensitive, the observation of a (viable) schistosome egg originating from a cervicovaginal surface has wider biological importance. Indeed, not to undertake inspection by light microscopy of CVL when there is an opportunity to do so, is missing out on both infection dynamic and environmental contamination indicators.

Thus, microscopy of CVL sediments has a clear adjunct role to better describe FGS, for it is both affordable and relatively straightforward to implement in low resource settings, can incriminate the direct presence of (viable) schistosome eggs within the genital tract (viewing their diagnostic morphology) and can provide a semi-quantitative measure that might track active infection before and after praziquantel treatment. Although there are cheap sampling devices, e.g. Delphi Screener, for commercial retail that enable collection of cervicovaginal self-lavage (Ndayisaba et al., 2013), genital self-swab methods have proven better useful and provide a more convenient clinical sample for molecular diagnosis across a range of parasites and pathogens (Delany et al., 2008; Sturt et al., 2020). Though it would be taxing to view schistosome eggs directly within the swab matrix itself, it is possible to incubate the swab within mineral water to release the free-swimming miracidium (J.R.S., *personal observations*). Obtaining miracidia in this way is still a useful adjunct but only applicable to genital swabs that do not contain preservative chemicals that interfere with the hatching process.

It is beyond the scope of this report to discuss the relative merits of clinical microscopy within sexual and reproductive health clinics (Martinez et al., 2024) alongside overcoming challenges in mainstreaming FGS surveillance and control across Africa (Mutapi et al., In review) but general diagnostic microscopy of CVL sediments has an ability to visualise other parasites present, e.g. *Trichomonas vaginalis*, see Figure 2A, as well as the presence of both red and white blood cells even with or without schistosome eggs evident. When schistosome eggs are present, however, white blood cells, most likely eosinophils, can often be seen to adhere to the egg’s shell surface which might help to explain some of the interesting cytokine profiles or taxonomic shifts in vaginal microbiota as described by others upon analysis of CVL (Sturt et al., 2021a, 2021b). We have also noted frank visual blood and cryptic blood, as revealed by reagent strips, which may have some gynaecological importance here, but how this correlates with FGS alone is not currently clear given its many clinical guises (Kjetland et al., 2012) and current or past associations with other genital tract co-infections (Sturt et al., 2022).

We suggest that the key reason to use microscopy of CVL sediments is firmly tied to current and future needs to genotype the schistosome infection more precisely either to incriminate whether these eggs or miracidia are from zoonotic species or are of a hybrid-species nature (Stothard et al., 2020a). By simply immersing the glass slide into a Petri dish containing mineral water, then removing the cover slip, it is possible to hatch and catch any viable schistosome miracidia onto FTA cards for later genotyping. This is a very simple field-friendly protocol for collecting several tens of miracidia with favourable possibilities for future scale-up. In our sample we were able to demonstrate the dominance of *S. haematobium* but also co-infections with *S. mattheei*, inclusive of a putative hybrid *S. haematobium* × *mattheei* miracidium. To devise effective future public health strategies to prevent and control FGS, especially when a component of these schistosome infections arises from zoonotic or hybrid-variants, carefully targeted molecular surveillance is needed. Looking ahead and if adopted more widely in research studies, it could provide clearer insights into the dynamics of genital tract disease in women at-risk from urogenital schistosomiasis.

## Data Availability

More detailed protocols and all data are available upon reasonable request to the corresponding author.
